# Comparison of the effect of dietary copper nanoparticles and one copper (II) salt on the copper biodistribution and gastrointestinal and hepatic morphology and function in a rat model

**DOI:** 10.1371/journal.pone.0197083

**Published:** 2018-05-14

**Authors:** Ewelina Cholewińska, Katarzyna Ognik, Bartosz Fotschki, Zenon Zduńczyk, Jerzy Juśkiewicz

**Affiliations:** 1 Department of Biochemistry and Toxicology, Faculty of Biology, Animal Sciences and Bioeconomy, University of Life Sciences in Lublin, Lublin, Poland; 2 Division of Food Science, Institute of Animal Reproduction and Food Research of the Polish Academy of Sciences, Olsztyn, Poland; University of Illinois, UNITED STATES

## Abstract

The aim of the study was to investigate the effect of two forms (CuCO_3_ (CuS); and Cu nanoparticles (CuNP)) and dosages (standard 6.5 mg/kg (H), half of the standard (L)) of additional dietary Cu administered to growing rats on gastrointestinal and hepatic function and morphology. Copper in the form of CuNP vs CuS caused lower Cu faecal/urinal excretion and increased Cu accumulation in the brain tissue. Hepatic high-grade hydropic degeneration and necrotic lesions were observed only in the CuNP-H animals. In the lower gut, the dietary application of CuNP stifled bacterial enzymatic activity of caecal gut microbiota and resulted in lower SCFA production. That diminishing effect of CuNP on caecal microbiota activity was accompanied by a relative increase in the secretion of glycoside hydrolases by bacterial cells. The results showed that in comparison to Cu from CuCO_3_, Cu nanoparticles to a greater extent were absorbed from the intestine, accumulated in brain tissue, exerted antimicrobial effect in the caecum, and at higher dietary dose caused damages in the liver of rats.

## Introduction

Copper (Cu) is an essential micronutrient for the functioning of living organisms. As a transition metal it has the ability to accept and donate electrons, so that it can be present in both oxidized (Cu^2+^) and reduced (Cu^2+^) forms. For this reason it is built into the active centres of numerous enzymes involved in metabolic processes crucial to the proper functioning of the organism [1,2], such as mitochondrial respiration, defence against free radicals, synthesis of neurotransmitters, collagen and elastin production, melanin synthesis, or iron metabolism [3,4,5]. However, excess copper is toxic, and therefore single-celled and multicellular organisms have developed precise, genetically-controlled mechanisms to preserve the homeostasis of this element [3,4].

Copper metabolism disorders lead to severe metabolic syndromes, such as Menkes disease or Wilson’s disease [2,6,7]. Moreover, they can contribute to the development of heart disease, diabetes and obesity, as copper is also involved in the biosynthesis of fatty acids and carbohydrates, which suggests that lipid biosynthesis is highly sensitive to changes in copper ion concentrations and that this element has a role in lipid metabolism [8,9].

Copper is absorbed from food into the small intestine (particularly in its first section, the duodenum) by endocytosis/pinocytosis, or is actively transported by the protein DMT1 and CTR1 [10]. It is then transported from the enterocytes into the bloodstream by the protein ATP7A [10]. In the plasma, copper ions are not present in their free state but in complexes with albumin, histidine and glutathione. In this form copper is distributed throughout the body with the blood [2]. The primary organ responsible for copper metabolism is the liver, which accumulates the greatest amount of this element [11]. Ceruloplasmin (Cp), a protein that binds copper atoms, is synthesized in the hepatocytes, and when released into the bloodstream it functions as the main factor maintaining the homeostasis of this microelement. It is worth noting that as much as 65–70% of the copper in the plasma is bound to Cp. Another protein produced in the liver is cysteine-rich metallothionein, which also binds excess copper in the body. Moreover, the liver is the site of production of bile, in which copper has the highest content of all microelements [11]. Excess Cu is also secreted to the bile, which then enters the intestine and is excreted from the body in the faeces. In this way 98% of copper is eliminated from the body. In the kidneys, during glomerular filtration, some of the copper passes to the primary filtrate, and in the proximal convoluted tubules it is reabsorbed and returned to the bloodstream. Ions that are not reabsorbed are excreted in the urine. About 2% of copper is eliminated in this manner [11].

In the last decade, advances in nanotechnology have enabled the development of methods for producing Cu on a nano scale (1–100 nm). Due to their unique properties distinguishing them from traditional structures, nanoparticles have found broad application in cosmetology, agriculture, the food and textile industries, and even in construction [12,13,14]. Metal nanoparticles—also copper, are also widely used in medicine, including in diagnostic imaging and in modern drug supply technology in targeted cancer therapy. The complex consisting of a therapeutics and a nanoparticle carrier after introduction into the patient’s body and the use of an external high gradient electromagnetic field is concentrated in well-defined tissues or organs [15]. Furthermore, the use of nanoparticle carriers has a positive effect on the prolonged release of the drug [16]. In addition, metal nanoparticles are very well suited for the treatment of adjuvant cancer therapy—hyperthermia. Devices commonly used for heating cancer cells in addition to destroying cancer cells also cause accidental damage to neighboring normal cells. Magnetic nanoparticles, in turn, act highly selectively, and their action is limited only to raising the temperature in the tumor area [15]. In addition, copper nanoparticles belonging to the transition elements, already used in small doses generate free radicals inducing DNA damage as a result, leading to apoptotic death of tumor cells, without affecting the normal cells negatively [17]. Copper nanoparticles are also successfully used in the treatment of bone defects resulting from bone regeneration disorders. Nanocopper in the form of copper-dopped mesosporus silica nanospheres have the ability to increase bone density due to the induction of osteogenic and angiogenic factors and inhibition of osteoclastogenic factors. What’s more, copper nanoparticles, showing a powerful antimicrobial effect, facilitates the healing of infected wounds resulting, for example, from an open fracture [18]. An important advantage of copper nanoparticles over nanoparticles of other precious metals, eg Au or Ag, is their decidedly lower price [17].

Besides the unquestionable benefits of nanotechnology, concerns about the potential toxicity of nanoparticles are increasingly raised [19–21]. It may be due to their high chemical reactivity, tendency to aggregate, porosity, and high affinity for a number of biological structures [22,23]. Due to their small size, nanometals have the ability to penetrate biological membranes, block ion channels, inhibit enzyme proteins, and interact with genetic material. These processes can lead to cell and tissue damage and generate oxidative stress with all of its consequences [24,25].

While experiments have recently been conducted to assess the effect of copper nanoparticles on the absorption of the element, liver metabolism, redox status, and livestock performance [10,26], knowledge of this issue remains insufficient and research results are very often inconsistent. It is also worth noting that research has most often been conducted in vitro using fixed cell lines (primarily murine macrophages and human dendritic cells), as well as on animals in completely different experimental models (different nanoparticle sizes and different routes and durations of administration).

In the present study it was hypothesized that substantial changes in rat gastrointestinal/hepatic function and morphology would follow the dietary replacement of CuCO_3_ with copper nanoparticles. To verify that hypothesis, copper digestibility, utilization and tissue distribution as well as small intestinal and liver histological parameters and caecal microbial activity were thoroughly examined.

## Material and methods

### Nanoparticles characterization

Copper nanoparticles were obtained from Sky Spring Nanomaterials, Inc. (Houston, TX, USA), with purity of 99.9%, particles 40–60 nm in size (nanopowder), spherical morphology, 0.19 g/cm^3^ bulk density, and 8.9 g/cm^3^ true density.

### Animal protocol and dietary treatments

All animal care and experimental protocols complied with the current laws governing animal experimentation in the Republic of Poland and by an ethical committee according to the European Convention for the Protection of Vertebrate Animals used for Experimental and other Scientific Purposes, Directive 2010/63/EU for animal experiments, and were approved by the Local Ethics Committee for Animal Experiments in Olsztyn, Poland (Permit Number: 68/2017).

Forty, healthy, male, albino Wistar rats (Han IGS Rat [Crl:WI(Han)]) aged 5 weeks with average body weight of 135 ± 10 g were randomly divided into 5 groups. Rats were housed randomly and individually in stainless steel cages under a stable temperature (21–22°C), relative humidity 50 ± 10%, a 12-h light-dark cycle, and a ventilation rate of 20 air changes per hour. All animals throughout the study were monitored daily by the vet for any abnormal rat’s behaviour in order to respect the humane endpoints in animal research. The rats were also carefully observed by trained technical staff to recognise any indicators of animal’s fear, distress, pain or anxiety. For 4 weeks, the rats had free access to tap water and semipurified diets, which were prepared and then stored at 4°C in hermetic containers until the end of the experiment (details in Table 1). The diets were modifications of a casein diet for laboratory rodents recommended by the American Institute of Nutrition. In the study, five experimental treatments were used to evaluate the effects of different levels of Cu in the diet as the mineral mixture (standard dosage 6.5 mg/kg diet, half the standard dosage, and additionally as a negative control no Cu in mineral mixture) and two Cu sources (CuCO_3_—commonly used in rodent laboratory diets; and Cu nanoparticles preparation, 40–60 nm).

**Table 1:** Composition of basal diet fed to rats, %.

Ingredient	Content
*Unchangeable ingredients*:	
Casein^a^	14.8
DL-methionine	0.2
Cellulose^b^	8.0
Choline chloride	0.2
Rapeseed oil	8.0
Cholesterol	0.3
Vitamin mix^c^	1.0
Maize starch^d^	64.0
*Changeable ingredient*:	
Mineral mix^e^	3.5
*Calculated content*:	
Crude protein	13.5

^a^Casein preparation: crude protein 89.7%, crude fat 0.3%, ash 2.0%, and water 8.0%.

^b^α-Cellulose (SIGMA, Poznan, Poland), main source of dietary fibre.

^c^AIN-93G-VM, g/kg mix: 3.0 nicotinic acid, 1.6 Ca pantothenate, 0.7 pyridoxine-HCl, 0.6 thiamin-HCl, 0.6 riboflavin, 0.2 folic acid, 0.02 biotin, 2.5 vitamin B-12 (cyanocobalamin, 0.1% in mannitol), 15.0 vitamin E (all-rac-α-tocopheryl acetate, 500 IU/g), 0.8 vitamin A (all-trans-retinyl palmitate, 500000 IU/g), 0.25 vitamin D-3 (cholecalciferol, 400000 IU/g), 0.075 vitamin K-1 (phylloquinone), 974.655 powdered sucrose.

^d^Maize starch preparation: crude protein 0.6%, crude fat 0.9%, ash 0.2%, total dietary fibre 0%, and water 8.8%.

^e^Changeable dietary ingredient in relation to copper level; mineral mixture with different Cu level originated from two sources (standard source CuCO_3_ and experimental Cu nanoparticles preparation), see Tables 2 and 3.

The rats were divided into following groups: the CuD group—during all four weeks of feeding the CuD rats as a negative control group (CONT) were administered a diet with mineral mix (MX) deprived of Cu (CuCO_3_ excluded from MX); the CuS-H group—the rats were fed a diet with standard mineral mixture (MX) resulting in 6.5 mg Cu (from CuCO_3_ in MX) per 1 kg of a diet during 4 weeks of feeding; the CuS-L group—the CuSalt-L rats were administered a diet with half the standard dosage (6.5 mg/kg) from CuCO_3_ in MX during 4 weeks of feeding; the CuNP-H group—the CuNano rats were administered a diet containing 6.5 mg/kg Cu from the Cu nanoparticles preparation at the standard concentration per 1 kg of a diet during 4 weeks of feeding; the CuNP-L group—the CuNano-L rats were fed a diet with half the standard dosage (6.5 mg/kg) from Cu nanoparticles preparation per 1 kg of a diet during 4 weeks of feeding (see Table 2). In order to keep the operator safe while preparing the experimental diets, the CuNP preparation was added to a diet not in mineral mixture, but as an emulsion along with dietary rapeseed oil. The detailed composition of mineral mixtures used in all experimental groups has been provided in Table 3.

**Table 2:** Experimental schema^a^ (provided copper dosage was calculated taking into account CuCO_3_ in MX or copper from Cu nanoparticles preparation).

Treatment	Weeks of feeding			
	1^st^	2^nd^	3^rd^	4^th^
CuD (CONT, without Cu in MX)	A diet with MX deprived of Cu (n = 8)			
CuS-H	A diet containing 6.5 mg/kg Cu from CuCO_3_ (n = 8)			
CuS-L	A diet containing 3.25 mg/kg Cu from CuCO_3_ (n = 8)			
CuNP-H	A diet containing 6.5 mg/kg Cu from Cu nanoparticles preparation (n = 8)			
CuNP-L	A diet containing 3.25 mg/kg Cu from Cu nanoparticles preparation (n = 8)			

n = 8, number of rats used in particular feeding period.

^a^Experimental groups: CONT = CuD—during all four weeks of feeding the Cu deficient rats were given a diet with MX deprived of Cu (CuCO_3_ excluded from MX); CuS-H—the rats were fed a diet with standard mineral mixture (MX) resulting in 6.5 mg Cu (from CuCO_3_ in MX) per 1 kg of a diet during 4 weeks of feeding; CuS-L—the rats were given a diet with half the standard dosage in mineral mixture (MX) resulting in 3.75 mg Cu (from CuCO_3_ in MX) per 1 kg of a diet during 4 weeks of feeding; CuNP-H—the rats were given a diet containing 6.5 mg·kg^-1^ Cu from Cu nanoparticles preparation per 1 kg of a diet during 4 weeks of feeding; CuNP-L—the rats were given a diet with half the standard dosage (6.5 mg/kg) from Cu nanoparticles preparation per 1 kg of a diet during 4 weeks of feeding.

**Table 3:** Composition of mineral mixtures (MX) used in experimental diets, g/kg.

	MX with standard Cu dosage^a^	MX with half standard Cu dosage^b^	MX deprived of Cu^c^
Calcium carbonate anhydrous CaCO_3_	357	357	357
Potassium phosphate monobasic K_2_HPO_4_	196	196	196
Potassium citrate C_6_H_5_K_3_O_7_	70.78	70.78	70.78
Sodium chloride NaCl	74	74	74
Potassium sulphate K_2_SO_4_	46.6	46.6	46.6
Magnesium oxide MgO	24	24	24
Microelements mixture^#^	18	18	18
Starch	213.62	213.62	213.62
^#^Microelements mixture, g/100g			
Ferric citrate (16,7% Fe)	31	31	31
Zinc carbonate ZnCO_3_ (56% Zn)	4.5	4.5	4.5
Manganous carbonate MnCO_3_ (44.4% Mn)	23.4	23.4	23.4
Copper carbonate CuCO_3_ (55.5% Cu)	1.85	0.925	0
Potassium iodate KJ	0.04	0.04	0.04
Citric acid C_6_H_8_O_7_	39.21 g	40.135	40.7

^a^given to CuS-H groups (4 weeks of feeding);

^b^given to CuS-L group (4 weeks of feeding);

^c^given to CuNP-H, CuNP-L groups (4 weeks of feeding); during 2–5 weeks of feeding the groups CuNP-H and CuNP-L were provided with appropriate amount of Cu from Cu nanoparticles preparation as an emulsion along with dietary rapeseed oil.

All physiological measurements were done for each animal separately (n = 8 for each group). During the study, the digestibility and utilization tests (balance tests) of copper (Cu) were carried out. After a 10 d preliminary period, faeces and urine were thoroughly collected for 5 d from all rats that were kept in balance cages (Tecniplast Spa, Buguggiate, Italy). The content of Cu in diets, drinking water, faeces and urine collected in the balance period was assayed using the methods described below. Experimental groups were additionally monitored for body weight gain and feed intake. At the end of the experiment, the rats were fasted for 24 hours and anaesthetized *i*.*p*. with ketamine and xylazine (K, 100 mg/kg BW; X, 10 mg/kg BW) according to the recommendations for anaesthesia and euthanasia of experimental animals. Anaesthetized animals were subjected to the time-domain nuclear magnetic resonance using the minispec LF 90II analyzer (Bruker, Karlsruhe, Germany) in order to determine the fat and lean body mass. The minispec transmits various radio frequency pulse sequences into soft tissues to re-orient the nuclear magnetic spins of the hydrogen and then detects radio frequency signals generated by the hydrogen spins from these tissues. The contrast in relaxation times of the hydrogen spins found between adipose tissue and water-rich tissues is used to estimate fat and lean body mass. Then, after laparotomy blood samples were taken from *caudal vena cava*, and finally the rats were euthanized by cervical dislocation. After that the small intestine, caecum, spleen, liver, heart, kidneys, brain, lungs, and thigh muscle were dissected and weighed.

### Copper analyses

Copper content in the samples of water, feed mixture, urine, faeces, brain, liver was determined by inductively coupled plasma optical emission spectrometry (ICP-OES). The Certified Reference Material NIST-1577C Bovine liver was used for quality control.

### Caecal digesta analyses

Caecal pH was measured directly in the intestine using a microelectrode and a pH/ION meter (model 301, Hanna Instruments, Vila do Conde, Portugal). Samples of caecal contents were used for immediate analysis (ammonia, dry matter, short-chain fatty acids (SCFA), while the rest of the caecal digesta was transferred to tubes and stored at -70°C. In fresh caecal digesta, ammonia was extracted, trapped in a solution of boric acid in the Conway’s dishes, and determined by direct titration with sulphuric acid. Dry matter of digesta was determined at 105°C. Caecal digesta samples were subjected to SCFA analysis using gas chromatography (Shimadzu GC-2010; Kyoto, Japan). The samples (0.2g) were mixed with 0.2 mL formic acid, diluted with deionised water and centrifuged at 7211 × g for 10 min. The supernatant was loaded onto a capillary column (SGE BP21, 30 m × 0.53 mm) using an on-column injector. The initial oven temperature was 85°C and was raised to180°C by 8°C /min and held there for 3 minutes. The temperatures of flame ionization detector and the injection port were 180 and 85°C, respectively. The sample volume for GC analysis was 1 μL. The concentrations of caecal putrefactive SCFAs (PSCFAs) were calculated as the sum of iso-butyric acid, iso-valeric acid and valeric acid. All SCFAs analyses were performed in duplicate. Pure acetic, propionic, butyric, iso-butyric, iso-valeric and valeric acids were obtained from Sigma Co. (Poznan, Poland), and their mix was used to create a standard plot and then to calculate the amount of single acids. This additional set of pure acids was included in each GC run of samples at five sample intervals to maintain calibration.

### Activity of microbial enzymes in caecal digesta

Bacterial enzyme activity was measured by the rate of *p*- or *o*-nitrophenol release from their nitrophenylglucosides according to the method described elsewhere [27] in fresh caecal digesta (α- and β-glucosidase, α- and β-galactosidase, β-glucuronidase). The following substrates were used: *ρ*-nitrophenyl-*α*-D-glucopyranoside (for *α*-glucosidase), and *ρ*-nitrophenyl-*β*-D-glucopyranoside (for *β*-glucosidase), *ρ*-nitrophenyl-*α*-D-galactopyranoside (*α*-galactosidase), *o*-nitrophenyl-*β*-D-galactopyranoside (*β*-galactosidase), and *ρ*-nitrophenyl-*β*-D-glucuronide (for *β*-glucuronidase). The reaction mixture contained 0.3 mL of a substrate solution (5 mM) and 0.2 mL of a 1:10 (v/v) dilution of the caecal sample in 100 mM phosphate buffer (pH 7·0) after centrifugation at 7211 × g for 15 minutes. Incubation was carried out at 37°C and *ρ*-nitrophenol was quantified at 400 nm and at 420 nm (*o*-nitrophenol concentration) after the addition of 2.5 mL of 0·25 M cold sodium carbonate. The enzymatic activity (*α*- and *β*-glucosidase, *α*- and *β*-galactosidase, and *β*-glucuronidase) was expressed as μmol product formed per an hour per g of digesta. In order to determine the total activity of selected caecal bacterial enzymes, including extracellular activity (see the procedure above) and intracellular activity, a caecal digesta sample diluted in phosphate buffer was mechanically disrupted by vortexing with glass beads (212–300 μm in diameter; four periods of 1 min with 1 min cooling intervals on ice) using the FastPrep^®^-24 homogenizer (MP Biomedicals, Santa Ana, Ca, US). The resulting mixture was centrifuged at 7211 g for 15 min at 4°C. The supernatant was used for the enzyme assay described above. Intracellular enzyme activity was calculated by comparing total enzyme activity with the activities of bacterial enzymes secreted into the intestinal environment, and it was expressed as μmol product (PNP or ONP, ρ-nitrophenol or o-nitrophenol, respectively) formed per hour per g of digesta. In order to prepare the calculation formulas, the model curves for PNP and ONP (PNP or ONP standard solution in a 100mM phosphate buffer pH 7.0, 40 mg/L) were used and appropriate equations obtained. Extracellular enzyme activity was determined as the rate of enzyme release, expressed as a percentage of total enzyme activity. All analyses were performed in duplicate.

### Histological examinations of the liver and jejunum

Samples of the liver and jejunum were cut in two lengthwise and fixed for 24 h in 5% formalin, pH = 7.2. Within 24 hours the fixed tissue fragments were passed through increasing concentrations of alcohol solutions, acetone and xylene into paraffin blocks in a tissue processor (Leica TP-20). Paraffin-embedded microscope sections 5 μm thick were stained with hematoxylin and eosin (HE staining). Morphometric evaluation of the liver, length of the villi and depth of the crypts was carried out using a computer-assisted microscopic image analysis system. The system includes a light microscope (Nikon Eclipse E600) with a digital camera (Nikon DS-Fi1) and a PC with image-analysis software (NIS-Elements BR-2.20, Laboratory Imaging). In each jejunum tissue slide 20 villi cut in two lengthwise and 20 crypts were measured. The length of the villi was measured from the tip to the base.

### Plasma biochemical analyses

Blood samples were collected carefully from *caudal vena cava* at the end of the study for plasma biochemical analysis. The plasma was immediately separated by centrifugation and stored at -70°C for further analysis. The activities of alanine aminotransferase (ALT), aspartate aminotransferase (AST), alkaline phosphatase (ALP), creatine kinase (CK), lactate dehydrogenase (LDH) and gamma-glutamyl transferase (GGT) were measured using an automatic biochemical analyzer (Plasma Diagnostic Instruments Horiba, Kyoto, Japan).

### Statistical analysis

The model assumptions of normality and homogeneity of variance were examined by the Shapiro-Wilk and Levene tests, respectively. To compare the CuD (mineral mix deprived of copper) group versus each experimental one, the data were subjected to the independent (unpaired) samples *t*-test procedure. In a model without the CuD group, the data were subjected to 2-way ANOVA to examine the main effects: F—Cu form effect (two types of Cu form: CuCO_3_ in mineral mix and Cu nanoparticles; S and NP treatments, respectively), D—Cu dietary dose effect (3.75 and 6.5 mg Cu per one kg of a diet; L and H treatments, respectively), and the interaction between these two factors (F × D). If the analysis revealed a significant interaction (P≤0.05), the differences among the respective treatment groups (CuS-H, CuS-L, CuNP-H, CuNP-L) were then determined with the Newman-Keuls post hoc test at P ≤ 0.05. The statistical analysis was performed according to the GLM procedure for Statistica 10.0 software (StatSoft Corp., Krakow, Poland). Treatment effects were considered to be significant at P≤0.05. All data were expressed as mean values with pooled SEM. SEM, standard error of the mean was calculated by using SD for all rats divided by square root of rat number (n = 40).

## Results

### Cu-deficient (CuD) dietary treatment vs treatments with CuCO_3_ and nano-Cu (studentized *t*-test procedure)

In the treatments in which the rats received CuCO_3_ or copper nanoparticles, at both the standard dose of 6.5 mg/kg (CuS-H and CuNP-H) and half of this dose, i.e. 3.25 mg/kg (CuS-L and CuNP-L), a significantly greater (*P*<0.001) total quantity of Cu was excreted with the faeces and urine in comparison to the CuD group (Table 4). It was due to significantly lower faecal Cu excretion in Cu-deficient rats in comparison to other groups, despite surprising effect revealing that the CuD rats were characterized by significantly higher quantity of Cu excreted with urine vs others. Therefore, the digestibility Cu index was significantly higher in the CuD group (vs all others groups). In the case of the retention (utilization) Cu index, which considers the loss of Cu in both faeces and urine, it was found to be significantly lower in rats CuD vs both CuS treatments and higher in comparison to the CuNP-L rats.

**Table 4:** Copper excretion patterns in the digestibility and utilisation test as well as Cu concentration in liver and brain tissues.

Treatment	Cu in diet (mg/kg)	Diet intake (g/5 d)	Cu intake from a diet (mg/5 d)	Total Cu intake^#^ (mg/ 5 d)	Cu in urine (mg 5/d)	Cu in faeces (mg/5 d)	Total Cu excretion (mg/5 d)	Cu utilisation (%)	Cu digestibility (%)	Cu in liver (mg/kg of tissue)	Cu in brain (mg/kg of tissue)
CuD	3.58	79.8	0.307	0.309	0.237	0.017	0.255	17.7	94.5	0.921	3.87
CuS-H	6.21^b^*	76.2	0.473*	0.475*	0.172*	0.263*	0.436*	7.88*	44.4*	2.74*	3.81
CuS-L	5.21^d^*	72.8	0.379*	0.391*	0.205*	0.147*	0.352*	9.89*	62.6*	2.78*	4.28
CuNP-H	6.29^a^*	78.0	0.490*	0.492*	0.135*	0.265*	0.401*	17.9	46.0*	2.36*	4.58
CuNP-L	5.29^c^*	78.7	0.416*	0.417*	0.145*	0.143*	0.289*	31.3*	65.8*	2.61*	4.63
SEM	0.002	3.566	0.020	0.019	0.015	0.012	0.020	3.783	1.669	0.299	0.258
Form Cu (F)											
S	5.71	74.5	0.426	0.433	0.189	0.205	0.394	8.88	53.5	2.76	4.04
NP	5.79	78.3	0.453	0.455	0.140	0.204	0.345	24.6	55.9	2.48	4.60
Dosage Cu (D)											
H	6.25	77.1	0.481	0.483	0.154	0.264	0.418	12.9	45.2	2.55	4.19
L	5.25	75.8	0.397	0.404	0.175	0.145	0.320	20.6	64.2	2.69	4.45
P value											
F effect	0.689	0.280	0.196	0.254	0.003	0.955	0.018	<0.001	0.168	0.360	0.015
D effect	<0.001	0.705	<0.001	<0.001	0.154	<0.001	<0.001	0.039	<0.001	0.624	0.230
F×D interaction	<0.001	0.564	0.640	0.814	0.456	0.821	0.476	0.118	0.630	0.725	0.340

*Means within the same column differ significantly from the control group at *P*≤0.05 as a result of independent *t*-test procedure.

^a-d^Means within the same column differ significantly (*P*≤0.05) as a result of Newman-Keuls mean comparison (only in the case of significant F×D interaction; analysis done only within CuS and CuNP treatments).

SEM = standard error of the mean (SD for all rats divided by square root of rat number, n = 40);

^#^Total Cu intake from a diet and water (Cu concentration in water administered to rats 0.0182 mg/L)

The treatment with CuD diet caused a significant decrease in hepatic copper concentration as compared to all other treatments, but there were no changes when the Cu concentration was considered.

The lack of copper in the mineral mix in the diet of the CuD rats had no negative effect on growth performance, which was comparable to that noted in the other experimental treatments (Table 5). A significant decrease in the body’s lean mass followed the CuNP-H treatment in comparison to the CuD rats.

**Table 5:** Dietary intake, final body weight (BW), proportion of body fat and lean mass, and relative weights of selected internal organ of rats.

Treatment	Diet intake (g)	BW (g)	Fat (% of BW)	Lean (% of BW)	Spleen^#^	Testes^#^	Liver^#^	Heart^#^	Kidneys^#^	Brain^#^	Lungs^#^
CuD	655	309	22.7	68.7	0.264	1.024	4.19	0.285	0.624	0.591	0.405
CuS-H	639	301	25.2	65.6^b^	0.284	1.07	4.09	0.266	0.616	0.587	0.376
CuS-L	620	298	24.5	66.2^a^^b^	0.268	1.06	4.14	0.271	0.636	0.617	0.383
CuNP-H	636	303	25.8	64.91^b^*	0.267	1.05	4.14	0.265	0.626	0.616	0.380
CuNP-L	638	303	22.9	68.60^a^	0.253	1.06	4.22	0.266	0.641	0.612	0.379
SEM	11.22	8.313	0.976	0.784	0.015	0.037	0.115	0.005	0.016	0.016	0.017
Form Cu (F)											
S	630	299	24.8	65.9	0.276	1.06	4.12	0.268	0.626	0.602	0.379
NP	637	303	24.3	66.8	0.260	1.06	4.18	0.265	0.634	0.614	0.379
Dosage Cu (D)											
H	638	302	25.5	65.3	0.275	1.06	4.12	0.266	0.621	0.601	0.378
L	629	300	23.7	67.4	0.261	1.06	4.18	0.268	0.638	0.615	0.381
P value											
F effect	0.470	0.470	0.554	0.213	0.210	0.836	0.564	0.598	0.628	0.403	0.981
D effect	0.402	0.620	0.047	0.004	0.265	0.998	0.572	0.585	0.260	0.341	0.749
F×D interaction	0.305	0.666	0.205	0.031	0.911	0.711	0.873	0.755	0.890	0.226	0.666

*Means within the same column differ significantly from the control group at *P*≤0.05 as a result of independent *t*-test procedure.

^a-d^Means within the same column differ significantly (*P*≤0.05) as a result of Newman-Keuls mean comparison (only in the case of significant F×D interaction; analysis done only within CuS and CuNP treatments). SEM = standard error of the mean (SD for all rats divided by square root of rat number, n = 40);

^#^g/100g BW.

In comparison to the CuD group, both dietary treatments with nano-Cu caused a significant decrease in intracellular and total activity of bacterial α-glucosidase in the caecum of rats (Table 6). As a result, the release rate of α-glucosidase in the caecal milieu, expressed as the percentage of extracellular enzymatic activity in relation to the total activity of that enzyme (sum of extracellular and intracellular activity), was significantly increased in groups CuNP-H and CuNP-L (*P*<0.05 vs CuD). In comparison to the CuD treatment, the released rate of bacterial β-glucosidase from bacterial cells into the caecal environment was significantly reduced in the CuS-H group and significantly increased in all other treatments (*P*<0.05). Irrespective of the Cu form and dose, a lower intracellular activity of bacterial α-galactosidase in the caecum followed all dietary treatments with additional Cu in comparison to the CuD rats (Table 7). As a result, the rate (% of total activity) at which that enzyme was released from bacterial cells into the caecal environment was significantly higher in both groups with nano-Cu than in group CuD. All treatments with Cu addition (CuCO_3_ or nano-Cu) were characterized by a decreased blood plasma ALP activity (*P*<0.05 vs CuD rats; Table 8).

**Table 6:** Activity of microbial enzymes in caecal digesta of rats (μmol/h/g fresh caecal digesta).

Treatment	α- glucosidase				β-glucosidase			
	Extra-	Intra-	Total	Release^a^	Extra-	Intra-	Total	Release^a^
CuD	14.4	2.85	17.2	83.3	1.43	1.01	2.44	59.0
CuS-H	15.7	2.48	18.2	86.3	1.71	1.04	2,75	31.8*
CuS-L	14.6	2.46	17.1	85.5	1.45	1.03	2.48	61.8*
CuNP-H	12.5	1.32*	13.8*	90.4*	1.44	0.35	1.79	81.9*
CuNP-L	12.3	1.41*	13.7*	89.6*	1.42	0.33	1.76	79.9*
SEM	0.758	0.302	0.751	1.948	0.261	0.179	0.388	3.879
Form Cu (F)								
S	15.2	2.47	17.6	85.9	1.58	1.03	2.61	46.8
NP	12.4	1.37	13.8	90.0	1.43	0.339	1.77	80.9
Dosage Cu (D)								
H	14.1	1.90	16.0	88.4	1.57	0.691	2.27	56.8
L	13.4	1.94	15.4	87.6	1.44	0.680	2.12	70.9
P value								
F effect	<0.001	<0.001	<0.001	0.031	0.533	<0.001	0.019	<0.001
D effect	0.268	0.891	0.268	0.670	0.550	0.947	0.658	0.786
F×D interaction	0.465	0.837	0.386	0.984	0.607	0.990	0.727	0.794

*Means within the same column differ significantly from the control group at *P*≤0.05 as a result of independent *t*-test procedure.

SEM = standard error of the mean (SD for all rats divided by square root of rat number, n = 40). Extra: extracellular activity; intra: intracellular activity; total: sum of extra- and intracellular activity;

^a^extracellular expressed as percentage of total (extra- + intracellular) enzymatic activity.

**Table 7:** Activity of microbial enzymes in caecal digesta of rats (μmol/h/g fresh caecal digesta).

Treatment	α-galactosidase				β-galactosidase				β-glucuronidase			
	Extra-	Intra-	Total	Release^a^	Extra-	Intra-	Total	Release^a^	Extra-	Intra-	Total	Release^a^
CuD	8.37	5.59	14.0	62.2	28.2	10.4	38.7	72.6	12.6	9.78	22.4	57.7
CuS-H	9.67	4.76*	14.4	68.3	31.3	11.3	42.6	72.9	11.5	10.4	21.9	53.6
CuS-L	8.50	3.85*	12.3	69.8	31.2	11.8	43.0	72.1	12.3	8.11	20.4	60.2
CuNP-H	7.75	2.69*	10.4	74.3*	27.0	5.75	32.8	82.9	11.5	6.45	18.0	64.9
CuNP-L	7.59	2.59*	10.2	73.7*	27.2	6.64	33.9	81.1	11.6	6.69	18.3	63.7
SEM	0.737	0.819	1.181	4.580	2.968	1.511	3.419	3.451	0.891	1.067	1.669	3.212
Form Cu (F)												
S	9.08	4.31	13.4	69.1	31.3	11.5	42.8	72.5	11.9	9.26	21.1	56.9
NP	7.67	2.64	10.3	74.0	27.1	6.19	33.3	82.0	11.6	6.57	18.2	64.3
Dosage Cu (D)												
H	8.71	3.73	12.4	71.3	29.2	8.51	37.7	77.9	11.5	8.43	19.93	59.2
L	8.04	3.22	11.3	71.7	29.2	9.22	38.4	76.6	12.0	7.40	19.4	61.9
P value												
F effect	0.041	0.013	0.002	0.234	0.131	<0.001	0.004	0.007	0.700	0.002	0.021	0.016
D effect	0.324	0.426	0.207	0.917	0.990	0.627	0.809	0.684	0.541	0.221	0.639	0.367
F×D interaction	0.449	0.528	0.325	0.797	0.958	0.897	0.914	0.885	0.632	0.132	0.457	0.189

*Means within the same column differ significantly from the control group at *P*≤0.05 as a result of independent *t*-test procedure. SEM = standard error of the mean (SD for all rats divided by square root of rat number, n = 40). Extra: extracellular activity; intra: intracellular activity; total: sum of extra- and intracellular activity;

^a^extracellular expressed as percentage of total (extra- + intracellular) enzymatic activity.

**Table 8:** Activity of selected enzymes in plasma of rats fed experimental diets (U/l).

Treatment	AST	ALT	ALP	GGT	CK	LDH
CuD	59.3	35.3	728	6.16	0.022	1140
CuS-H	52.2	36.1^b^	629*	6.35	0.024	1113
CuS-L	64.1	40.5^a^^b^	683*	5.19	0.023	1167
CuNP-H	57.3	32.6^b^	654*	5.93	0.019	1119
CuNP-L	61.7	50.5^a^	683*	5.69	0.026	1164
SEM	4.338	3.520	26.78	0.524	0.006	65.97
Form Cu (F)						
S	58.2	38.3	656	5.770	0.023	1140
NP	59.5	41.6	669	5.809	0.023	1141
Dosage Cu (D)						
H	54.74	34.3	641	6.14	0.022	1116
L	62.89	45.5	683	5.44	0.025	1166
P value						
F effect	0.725	0.297	0.539	0.923	0.889	0.989
D effect	0.037	0.001	0.048	0.097	0.607	0.449
F×D interaction	0.332	0.038	0.553	0.261	0.489	0.946

*Means within the same column differ significantly from the control group at *P*≤0.05 as a result of independent *t*-test procedure.

^a-b^Means within the same column differ significantly (*P*≤0.05) as a result of Newman-Keuls mean comparison (only in the case of significant F×D interaction; analysis done only within CuS and CuNP treatments).

ALT, alanine aminotransferase; AST, aspartate aminotransferase; ALP, alkaline phosphatase; CK, creatine kinase; LDH, lactate dehydrogenase; GGT, gamma-glutamyl transferase. SEM = standard error of the mean (SD for all rats divided by square root of rat number, n = 40).

### Two-way ANOVA results (a model without the CuD group): Cu form and dose effects and their interaction

Two-way ANOVA results showed that irrespective of the form of Cu (F), the dietary low (L; half of the standard recommended dosage of Cu in mineral mix) dose of additional copper significantly reduced the amount of Cu excreted with faeces, thus the L treatment decreased total Cu excretion and caused a significant increase in the values of digestibility and utilization Cu indexes (Table 4). In regard to the F main effect, the dietary application of Cu nanoparticles significantly decreased the amount of Cu excreted with urine, same did with total Cu excretion, thus caused a significant increase in the utilization Cu index, which considers the loss of Cu in both faeces and urine. Increased Cu concentration in brain tissue followed the NP treatment in comparison to the S one.

**Fig 1:**
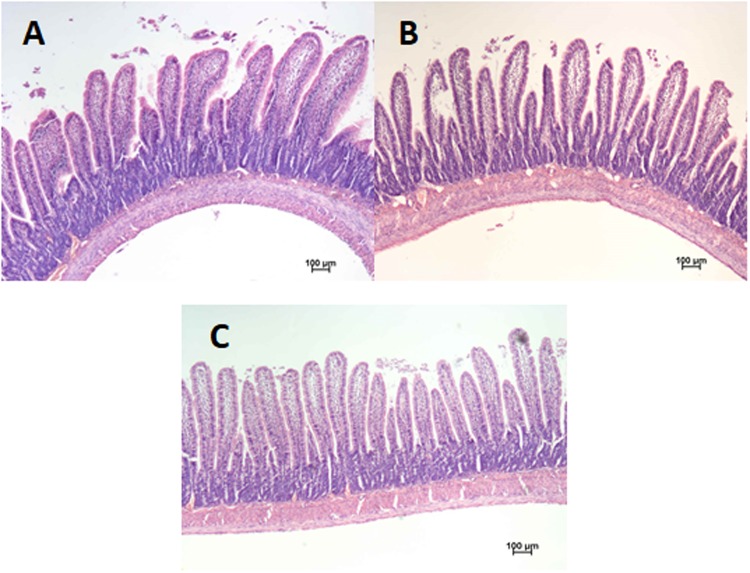
Morphological effects of different Cu sources on rat intestine—(A) CuD group; (B) CuS-H, CuS-L i CuNP-L groups; (C) CuNP-H group (magnification 10×).

The two-way ANOVA revealed that the L treatment caused a significant reduction in the percentage of body’s fat mass (*P* = 0.047 vs H), irrespectively of the Cu form (Table 5). The Cu form by dose interaction showed significant increase in the percentage of body’s lean mass in rats fed CuNP-L diet in comparison to their counterparts fed a diet with higher dose of Cu nanoparticles. Such significant effect was not observed within rats receiving copper as CuCO_3_ in mineral mixture. The NP treatment was accompanied with a significant increase in the jejunal crypt’s depth in comparison to the S treatment (*P*<0.001; Table 9). As shown by a significant Cu form by dose interaction (F×D), a lower dose of copper in the form of CuCO_3_, but not as nanoparticles, caused significant reduction in the length of jejunal villi. Additionally, the CuNP-L rats were characterized by similar length of villi in the jejunum as in the CuS-H animals. On the Fig 1 is shown the morphological effects of different Cu sources on rat intestine.

**Table 9:** The relative weight of small intestine with contents, measurements of jejunal villi and crypts in experimental rats as well as basic caecal indices.

		Small intestine			Caecum		
Treatment	Mean length of villi of the jejunum (μm)	Mean depth of crypt of the jejunum (μm)	Full mass (g/100g BW)	Tissue (g/kg BW)	Digesta (g/kg BW)	Ammonia (mg/g)	pH of digesta
CuD	562	154	2.29	0.174	0.544	0.254	7.06
CuS-H	555^a^	133	2.16	0.175	0.556	0.245	7.09
CuS-L	486^b^	136	2.17	0.172	0.564	0.258	6.98
CuNP-H	525^a^^b^	157	2.25	0.188	0.523	0.251	7.18
CuNP-L	564^a^	168	2.17	0.179	0.564	0.239	7.16
SEM	16.55	6.290	0.060	0.010	0.044	0.019	0.096
Form Cu (F)							
S	520	134	2.16	0.174	0,560	0.252	7.03
NP	545	162	2.21	0.184	0.543	0.245	7.17
Dosage Cu (D)							
H	540	145	2.20	0.182	0.539	0.248	7.14
L	525	152	2.17	0.176	0.564	0.249	7.07
P value							
F effect	0.149	<0.001	0.319	0.318	0.649	0.699	0.118
D effect	0.363	0.205	0.426	0.554	0.486	0.972	0.418
F×D interaction	0.003	0.482	0.309	0.742	0.640	0.422	0.593

^a-b^Means within the same column differ significantly (*P*≤0.05) as a result of Newman-Keuls mean comparison (only in the case of significant F×D interaction; analysis done only within CuS and CuNP treatments).

SEM = standard error of the mean (SD for all rats divided by square root of rat number, n = 40).

The two-way ANOVA showed that irrespective of applied Cu dose, the dietary application of copper in the nanoparticles form significantly decreased total caecal activity, including extra- and intra-cellular activities, of bacterial α-glucosidase and α-galactosidase in the caecum of rats, in comparison to the S treatment (Tables 6 and 7).

In the case of bacterial β-glucosidase, β-galactosidase, and β-glucuronidase, in the NP treatment a significant decrease in caecal enzymatic activity was observed in relation to intracellular and total activities of those enzymes. As a result of the aforementioned changes, the calculated release rate of an enzyme into the caecal milieu was significantly elevated in rats NP (vs rats S) in the case of all examined enzymes, except α-galactosidase. The dietary treatment with Cu-nanoparticles (NP) significantly decreased the caecal concentration of acetic and propionic acids, as well as total SCFA as compared to the S treatment (Table 10). The SCFA profile was only affected by the Cu form; the nanoparticles application significantly decreased the acetate proportion as related to total SCFA (*P* = 0.003 vs S).

**Table 10:** Short-chain fatty acid (SCFA) concentration and profile in the caecal digesta of rats.

Treatment	Concentration (μmol/g fresh digesta)								Profile (% of total SCFA)		
	C2	C3	C4i	C4	C5i	C5	PSCFA	SCFA	C2	C3	C4
CuD	49.0	11.6	1.59	6.93	0.898	1.28	3.76	71.4	68.6	16.4	9.70
CuS-H	52.7	11.1	1.36	7.07	1.09	1.28	3.73	74.7	70.7	14.9	9.40
CuS-L	49.6	11.0	1.24	7.70	0.967	1.21	3.42	71.7	69.1	15.5	10.7
CuNP-H	40.5	9.72	1.50	6.83	1.09	1.16	3,74	60.8	66.6	16.0	11.2
CuNP-L	41.6	10.1	1.20	6.79	1.05	1.06	3.30	61.8	67.1	16.4	11.1
SEM	2.926	0.683	0.147	0.618	0.143	0.117	0.327	4.002	1.033	0.599	0.697
Form Cu (F)											
S	51.2	11.1	1.30	7.38	1.03	1.24	3.57	73.2	69.9	15.2	10.1
NP	41.0	9.91	1.35	6.81	1.07	1.11	3.52	61.3	66.8	16.2	11.2
Dosage Cu (D)											
H	46.6	10.4	1.43	6.95	1.09	1.22	3.74	67.7	68.6	15.4	10.3
L	45.6	10.6	1.22	7.25	1.01	1.13	3.36	66.7	68.1	15.9	10.9
P value											
F effect	<0.001	0.038	0.712	0.299	0.685	0.202	0.855	<0.001	0.003	0.058	0.106
D effect	0.646	0.788	0.120	0.585	0.404	0.428	0.174	0.751	0.566	0.376	0.378
F×D interaction	0.348	0.648	0.501	0.538	0.665	0.869	0.811	0.516	0.255	0.824	0.305

SEM = standard error of the mean (SD for all rats divided by square root of rat number, n = 40). PSCFA, putrefactive SCFA (the sum of iso-butyric, iso-valeric and valeric acids); acids: C2, acetic; C3, propionic; C4i, iso-butyric; C4, butyric; C5i, iso-valeric; C5, valeric.

Irrespective of the form of Cu, the L dose caused a significant increase in plasma AST and ALP activities (*P* = 0.037 and *P* = 0.048 vs H, respectively; Table 8). As shown by a significant Cu form by dose interaction (F×D), a lower dose of copper in the form of nanoparticles, but not as CuCO_3_, caused a significant increase in plasma ALT activity. Interestingly, both H groups (CuS-H and CuNP-H) were characterized by lower ALT activity vs CuNP-L rats.

### Histopathological evaluation of the liver

**Fig 2:**
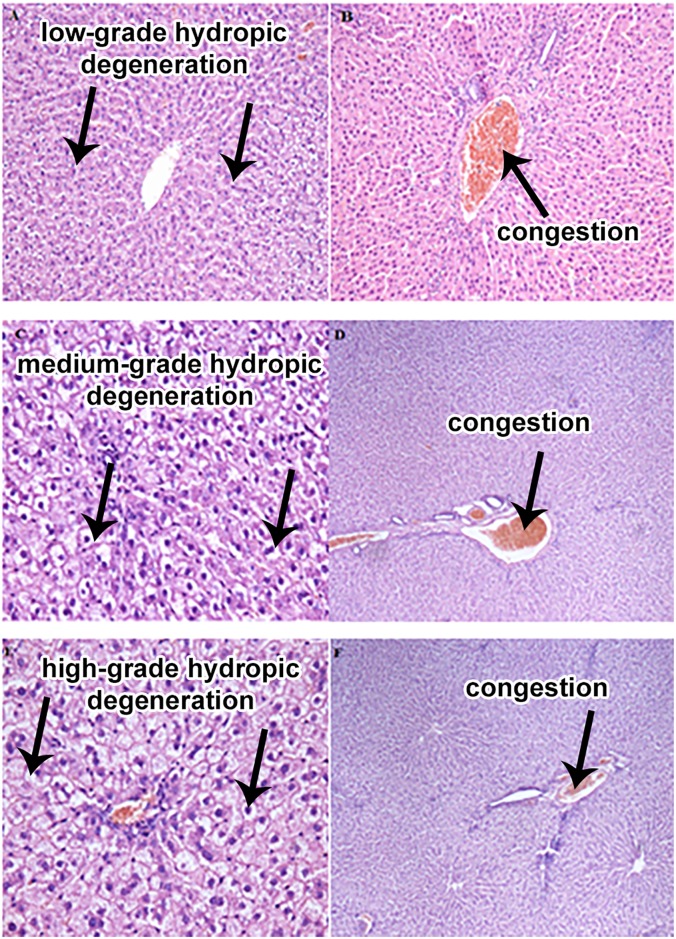
Morphological effects of different Cu sources on rat liver. (A-B) CuD group showing low-grade hydropic degeneration (A, magnification10×; B, magnification 10×); (C-D) CuS-H, CuS-L i CuNP-L groups showing medium-grade hydropic degeneration (C, magnification 20×; D, magnification 4×); (E-F) CuNP-H group showing high-grade hydropic degeneration and necrotic lesions (E, magnification; 20×; F, magnification 4×). Experimental groups: see Table 2.

Histopathological evaluation of the liver (Fig 2A–2F) showed passive congestion in the rats from all experimental treatments. In addition, low-grade hydropic degeneration was observed in the livers of the rats from the CuD treatment (Fig 2A and 2B). Medium-grade hydropic degeneration was noted in the livers of the rats receiving CuS-H, CuS-L or CuNP-L (Fig 2C and 2D), and high-grade hydropic degeneration as well as necrotic lesions in the rats from the CuNP-H treatment (Fig 2E and 2F). The criteria for classifying hydropic degeneration was the degree of cell damage.

## Discussion

The study showed that irrespective of the additional Cu dose, the CuS or CuNP rats excreted more copper in the urine and faeces and, interestingly, those treatments had lower Cu digestibility with respect to the rats whose diet had no copper supplement in the mineral mix. Additionally, copper from the CuS treatment (CuCO_3_ in the mineral mix) was excreted with the urine and faeces to a greater extent than copper in the form of CuNP. These results suggest that the copper available in the dietary components (casein, starch, oil, etc.) was well absorbed by the organism of the rats and to a large extent covered the dietary Cu requirement. This is confirmed by the lack of differences between groups for parameters such as diet intake, body weight, and relative weight of selected internal organs. Similarly, Arnal et al. [28] fed rats a diet with no Cu or containing copper carbonate in the amount of 7 ppm (control), 35 ppm or 43 ppm for 30 d and found that increased copper in the diet resulted in increased excretion of the element in the faeces. In the same experiment, the authors found that copper administered in the amount of 7 ppm by the intraperitoneal route was excreted to a greater extent than the same amount of Cu administered orally. A dietary copper deficit did not cause excessive faecal excretion of this element (a successive decrease in Cu content in the faeces was observed as the duration of the experiment increased). Lee et al. [29], following administration of Cu in the form of nanoparticles (CuNPs—25 nm) or microparticles (CuMPs—14–25 μm) in the amount of 100, 200 or 400 mg/kg per d to 7-week-old Sprague-Dawley rats orally via a tube for 28 d, found that in the treatments with CuNPs the amount of urinal Cu increased with the amount of Cu in the diet. Lee et al. [26], in a study on 8-week-old male Sprague-Dawley rats receiving CuNPs or Cu in ionic form at a dose of 500 mg/kg BW, observed that Cu administered in ionic form was excreted significantly more in the urine, while CuNP in the faeces. Considering present and the above mentioned studies, it may be concluded that both the copper source (from natural dietary components or external substitution—in our case from CuCO_3_ or as nanoparticles) and the dietary Cu concentration are of paramount importance for its digestibility and utilization rate.

The varied and sometimes contradictory results for the absorption, biodistribution and excretion of Cu can undoubtedly be explained both by different routes of administration and by differences in the physicochemical properties of the copper forms used, which vary in terms of zeta potential, specific surface area and hydrodynamic diameters. Cu microparticles introduced orally in the form of carbonate dissociate into Cu^+2^ ions in the acidic environment of the gastrointestinal tract and are absorbed by various mechanisms in the enterocytes [10]. Cu^+2^ ions that are not absorbed in the small intestine reach further sections of the gastrointestinal tract where the pH is about 6.8–7.8. It is very likely that in alkaline pH, where OH^-^ predominates, Cu^+2^ ions form copper hydroxides (Cu(OH)_2_ that are not absorbed and are excreted in the faeces. Copper nanoparticles are very well oxidized to ionic forms in an acidic environment, as indicated by the high positive zeta potential reported by Lee et al. [26], whereas in an alkaline environment they undergo agglomeration, as indicated by the large hydrodynamic diameter [26].

The physiological solubility and physicochemical properties of the used dietary copper forms can affect their absorption and accumulation, then the biological response of the organism, and the amount of Cu excreted. The higher plasma ceruloplasmin activity in rats treated with CuNP in our further research [30] and the greater urinal/faecal Cu excretion in the CuS rats suggested that dietary Cu administered as CuNPs was better dissolved than CuS in an acidic environment and probably better absorbed in the digestive tract. The higher plasma ceruloplasmin activity in the rats treated with CuNP *vs* CuS indicated that more Cu ions dissociated from CuNP than from CuS and were absorbed into the systemic circulation and then bound to ceruloplasmin. Literature data indicate that 60–70% of the copper in plasma is bound to ceruloplasmin, 10–30% to transcuprein and 15–20% to albumin [2].

There is little knowledge how the host’s gastrointestinal tract responds to the dietary presence of copper nanoparticles. It is well known that great proportion of ingested nutrients and non-nutrients are absorbed (after digestion processes) in the small intestine and then via portal vein reach the liver. Hepatic Kupffer cells have been shown to be of paramount importance in nanoparticles elimination [31]. Very small metal nanoparticles (2 nm) are believed to be filtrated out of the liver through the kidneys, but bigger ones (40 nm) are retained in the Kupffer cells. In our study, the dosage and source of additional Cu had no effect on Cu accumulation in the liver. But comparison of those experimental treatments to control CuD rats revealed that the copper from CuCO_3_ or administered as nanoparticles accumulated to a greater extent (percentage of ingested Cu) than Cu originating from diet components, i.e. casein, cellulose, rapeseed oil, maize starch and others. Taking into account the results obtained in the present study, it should be stressed that copper nanoparticles more easily crossed the blood-brain barrier and were accumulated in the brain tissue as compared to copper from CuCO_3_, irrespective of the dose of dietary Cu added. Montenegro et al. [32] showed that nanoparticles of sizes 33–63 nm successfully crossed a blood-brain barrier *in vitro* model with the aid of transcellular pathway. Skalska et co-authors [33] observed that a low dose of nano-silver generated oxidative stress in rat’s brain but not in liver tissue, thus the latter tissue seems to be less vulnerable to nanoparticles than other organs. Many recent studies conducted on laboratory animals have shown that the addition of copper, primarily in the form of carbonate or sulphate, results in a dose-dependent accumulation of this element in the liver, brain and kidneys [28,34–41]. Lee et al. [26] found higher Cu levels in blood and organs (liver, kidneys and spleen) of the rats from the CuNP treatments *vs* rats receiving ionic Cu. Liu et al. [42] noted Cu accumulation in the liver and kidneys of mice treated with nasal-instilled CuNPs (23.5 nm) or CuMPs (17 um) at a dose of 40 mg/kg BW. Lee et al. [29], following administration of Cu nanoparticles (CuNPs—25 nm) or microparticles (CuMPs—14–25 μm) observed higher Cu concentration in the organs (liver, kidneys, spleen, brain, lungs and heart) of rats treated with CuNP in comparison to the CuMP treatments.

Our study showed that the absence of Cu supplementation in the rat diets had no negative effect on growth performance. Hence it can be assumed that copper metabolism was not impaired in the rats from any of the experimental treatments. Seol et al. [40] observed no effect of 11-week dietary supplementation with copper carbonate at doses of 6.36 mg Cu/kg or 0.93 mg Cu/kg in the diet of rats. According to other authors [34,43], Cu added (in the form of sulphate or carbonate) to the diet of rats improves their growth performance and reduces feed intake. Other research has shown that excessive doses of Cu in the diet of laboratory animals resulted in a deterioration of growth performance [38].

In our study, plasma ALP activity in rats receiving a diet without copper supplementation was higher than in rats whose diet contained additional copper. Interestingly, reducing the dosage of dietary copper (both CuS and CuNP) increased the activity of plasma AST, ALT and ALP. Given the opposite data presented by numerous authors [29,44,45] the results of our study were surprising and difficult to interpret, especially as the values of some of the parameters in the CuD (Cu-deficient) group were similar to those obtained in the high-dosage treatment while the values of others were similar to those noted in the low-dosage treatment. Similarly, Shukla et al. [46], administering 1 mg Cu/kg BW in the form of CuSO_4_ to Wistar rats orally for 16 weeks observed a significant decrease in ALT activity in both the blood and the liver, but no effect was on AST activity. Lee et al. [26] stressed that copper nanoparticles, having a much larger specific surface area than macro- or microparticles, can cause completely different biological reactions. Mohammadyari et al. [45] administered CuO nanoparticles (50 nm) to Wistar rats by intraperitoneal injection at dosages of 0, 100, 200 or 400 ppm for 15 d and observed a significant increase in serum AST, ALT and ALP activity only in the rats from the 400 ppm CuO-nano treatment as compared to 0 ppm. In a study in which rat diets were supplemented with CuNPs or CuMPs at doses of 100, 200 or 400 mg Cu/kg BW per d, AST, ALT, ALP and LDH activity increased with the dose of Cu [29]. Studies in which rats received Cu in the form of carbonate or sulphate also revealed a dose-dependent increase in the activity of liver enzymes AST, ALT, ALP, GGT and SDH [37,38,41].

In our study, passive congestion was observed in the livers from all experimental groups. Passive congestion of the liver is often a result of circulatory disorders. In the case of animal euthanasia this may be a variant of the norm. Low-grade hydropic degeneration was noted in the livers of the rats whose diet was not supplemented with copper. Medium-grade hydropic degeneration was observed in the livers of the rats whose diet included copper nanoparticles at a dose of 3.25 mg/kg or copper carbonate at a dose of 3.25 mg/kg or 6.5 mg/kg. High-grade hydropic degeneration and necrotic lesions were found in the livers of the rats receiving copper nanoparticles at a dose of 6.5 mg/kg. However, the changes may just be too normal glycogen and lipid accumulation, rather than a degenerative response. In already mentioned study by Liu et al. [42], only the highest dose of nanoparticles, i.e. 40 mg/kg BW, caused pathological hepatocyte necrosis. In another study [47], following administration of 0.5 ml of saline solution containing 0, 5, 10 or 100 mg/kg BW copper oxide (II) nanoparticles (CuO, 10–15 nm) to Wistar rats by injection for 7 d, histopathological examination of the liver revealed vasculature in the central veins and portal triad vessels in all CuO-nano groups. Cisternas et al. [48] fed Sprague-Dawley rats a diet containing less than 10 ppm of Cu or a diet containing 1,200 ppm of Cu in the form of CuSO_4_ for 16 weeks. Histopathological examination with a light microscopic showed no pathological changes in either the control or experimental rats, although a few animals receiving excess Cu were found to have benign areas of inflammatory cell accumulation with isolated necrotic lesions. However, histopathological examination under an electron microscope revealed ultrastructural changes in the group receiving the highest dose of Cu, i.e. lysosomal inclusions and changes in the nuclei (irregularly shaped cell nuclei containing condensed chromatin and increased occurrence of pyknotic nuclei following prolonged exposure), and in the mitochondria (increased abundance of mitochondria and size polymorphism). Other studies conducted on rats and mice showed that long-term exposure to excessive Cu doses can cause multifocal hepatitis, necrosis of individual cells, multiple haemorrhages, portal fibrosis, proliferative nodules, hemosiderin deposition, necrosis, and apoptosis, and may lead to enlargement or atrophy of the liver [26,39–41,49,50]. The formation of vacuoles inside hepatocytes allows for the delimitation of harmful factors to the effects of which the liver is exposed, from important cellular elements. As a result of the development of this defence mechanism, it is possible to reduce the functioning of this organ [51]. The criterion for the degree of hepatocyte cell damage suggests that excess CuNP can accumulate in hepatocytes and thus activate the vacuolation process. What’s more, as the amount of CuNP increases, the volume of vacuoles increases. Its excessive growth may, however, limit the normal position of the cell nucleus negatively affecting the cell, and even leading to its death [52].

In our study, cryptic depth in the jejunum of rats receiving a diet with 3.25 mg/kg or 6.5 mg/kg CuNP was greater than in the rats whose diet contained the same amount of CuS. Tomaszewska et al. [53], after 12 weeks of feeding Wistar rats a diet containing CuSO_4_ at a dose of 5 mg/kg BW (100% of the daily requirement) or adding a Cu-glycine chelate in the amount of 0.025, 0.01875 or 1.0125 mg Cu/l to their drinking water (meeting 100%, 75% or 50%, respectively, of the daily requirement for Cu), found that the use of Cu-glycine at a dose meeting 100% of the Cu requirement reduced the thickness of the muscular and submucosal layers and the depth of the crypts in the small intestine as compared to the control group receiving the standard dose and form of Cu. Han et al. [54], feeding Sprague-Dawley rats a diet supplemented with 80 mg/kg BW CuSO_4_, 80 mg/kg BW chitosan, 80 mg/kg BW copper chitosan-coated nanoparticles (121.9 nm) or 160 mg/kg BW chitosan-coated nanoparticles, found that the villus height in the small intestinal mucosa of the rats receiving both doses of copper nanoparticles increased significantly as compared to the control and the groups receiving 80 mg/kg BW of CuSO_4_ and chitosan. The authors found that application of lipid-coated copper nanoparticles led to favourable changes in the intestinal morphology.

Selected parameters of caecal function could be useful indicators for assessing dietary protein digestion in the small intestine. The movement of undigested dietary protein from the small intestine to the caecum is the most frequent cause of high levels of caecal ammonia and putrefactive SCFAs. Putrefactive SCFAs, including iso-butyric, iso-valeric and valeric acids, are produced mostly during the fermentation of amino acids in intestinal digesta or are synthetized by bacteria in the caecum and colon [55]. Ammonia in the large intestine could be derived from blood urea or from dietary protein that was not digested in the upper gastrointestinal tract [56]. In view of the research hypothesis, the concentrations of caecal ammonia and putrefactive SCFAs in the caecum were affected neither by Cu deprivation of dietary mineral mix nor by partial or complete substitution of CuCO_3_ with Cu-nanoparticles.

The digestive system of rats is highly specialized, and a large caecum support the utilization of fibrous materials by microbial degradation. These processes provide the host with additional energy, mainly through the production of SCFAs. In the present study, treatment NP with nanoparticles application was characterized by decreased caecal concentrations of SCFAs, in particular acetic and propionic acids.

Many authors investigated the effects of different dietary treatments on large gut microbiota by counting bacteria and their products or by measuring their enzymatic activity. In this study, bacterial counts were not measured; instead, we decided to determine the concentrations and profile of bacterial fermentation end-products (ammonia, SCFAs) and the activity of selected bacterial enzymes in the caecal digesta. According to Rowland [57], although bacteriological investigations (e.g. identification of selected groups) are useful in describing the basic ecology of the gut, they are of less value in studies of metabolism and nutrition. In an alternative approach, the microbiota are regarded as a separate “organ”, and the associated health consequences for the host can be assessed by selecting microbial enzyme activity or metabolic endpoints that result in compounds with potentially toxic or beneficial effects. In our study, an analysis of bacterial enzymatic activity in the caecal digesta revealed that lower SCFA concentration upon Cu nanoparticles could be attributed to significantly diminished bacterial total enzymatic activity, including both extracellular and intracellular activities, relative to treatment with CuCO_3_. The extracellular activity of bacterial enzymes has direct implications for the rate at which nutrients and non-nutrients undergo microbial digestion in the large intestine. Extracellular enzyme activity is influenced by the type and counts of bacterial species present in the digesta and by the rate of enzyme secretion by bacterial cells [58]. With regard to the research hypothesis, a higher release rate of glycoside hydrolases could be an important adaptive mechanism of intestinal bacteria which enables them to obtain additional energy from the large intestine through fermentation in the situation when Cu nanoparticles seem to significantly reduce bacterial enzymatic activity and probably caecal bacterial count. Mahapatra et al. [59] observed antibacterial effect of CuO nanoparticles towards some strains of *Klebsiella*, *Salmonella* and *Shigella* which was attributed to the crossing of nanoparticles through the bacterial cell membranes thus damaging intracellular enzymes of microbiota. It has been reported that nanoparticles may limit microbiota count via passing through nanomteric pores exist in the membranes of most bacteria [60].

In the available literature there are mixed outcomes when Cu nanoparticles supplemented to feed and their effects on large gut microbiota are taken into consideration [61]. Copper silicate nanoparticles, administered to chickens at a dose of 2 ppm, were found to positively modulate intestinal bacteria by increasing counts of *Lactobacillus species* and decreasing *E*. *coli* [62]. The results obtained in our study are closer to other scientific findings reported a strong antibacterial characteristics of metal nanoparticles towards intestinal microbiota and its metabolic activity [63,64]. Pietroiusti et al. [65] stated that in available literature the dominant view is that engineered nanomaterials rather cause adverse effects mediated by microbiome. That processes may occur through the direct killing of microbiota or through alternations of their function. In regard to the accepted hypothesis, our study clearly showed that in comparison to CuCO_3_ application both applied nano-Cu doses effectively reduced enzymatic activity of microbiota in the caecum thus decreasing SCFA concentration in the intestinal digesta. Das et al. [66] showed that the effect of Ag nanoparticles on intestinal bacteria was qualitatively different to that exerted by silver ions. Moreover, it has been observed that the smaller metal nanoparticles are the stronger their antibacterial effects [67]. That effect of nanoparticles is proposed to be originated from free metal ion toxicity arising from its dissolution from surface of the nanoparticles and/or oxidative stress of the nanoparticles [63].

## Conclusions

The results of this study indicate that dietary mineral mixture deprivation of copper seems to be of less importance to maintenance intestinal functioning in comparison to the dietary switch from CuCO_3_ to Cu nanoparticles. This substitution did exert a significant effect on Cu urine excretion and Cu retention in the body. The addition of Cu to the diet of rats, irrespective of the form and dose, contributed to greater excretion of this element in the faeces and urine and to lower digestibility than in the rats whose diet was not supplemented with Cu. Copper in the form of CuNP was better absorbed in the digestive tract than CuS, as indicated by its lower excretion in the urine and faeces and its greater accumulation in the brain. It should be stressed that the higher dosage of CuNP resulted in the occurrence of high-grade hydropic degeneration and necrotic lesions in the livers of the rats. The application of CuNP significantly stifled bacterial enzymatic activity of caecal gut microbiota and resulted in lower SCFA production. But the observed diminishing effect of CuNP on microbiota metabolic activity was accompanied by a relative increase in the secretion of glycoside hydrolases by bacterial cells in the caecum of rats. This seems to be one of the physiological mechanisms that enables to optimize nutrient absorption in the large intestine.
